# Identification and characterization of pumicyclin A, a novel circular bacteriocin from *Bacillus pumilus* with a dispensable leader peptide

**DOI:** 10.1128/mbio.01188-26

**Published:** 2026-06-24

**Authors:** Fangfang Liu, Xiaohui Huang, Paula M. O’Connor, Felipe Miceli de Farias, Colin Buttimer, Colin Hill, Oscar P. Kuipers, R. Paul Ross

**Affiliations:** 1 APC Microbiome Ireland, University College Cork 507057, Cork, Ireland; 2 Department of Molecular Genetics, University of Groningen 207104, Groningen, the Netherlands; 3 Teagasc Food Research Centre, Moorepark 34344 , Fermoy, Ireland; 4 School of Microbiology, University College Cork School of Microbiology 219553, Cork, Ireland; University of California, Berkeley, Berkeley, California, USA

**Keywords:** *Bacillus pumilus*, circular bacteriocins, pumicyclin A, bioactivity, heterologous production, mutagenesis

## Abstract

Foodborne pathogens such as *Listeria monocytogenes* and *Bacillus cereus* continue to pose serious threats to global food safety, driving demand for natural, consumer-friendly preservation strategies. Circular bacteriocins, a class of ribosomally synthesized antimicrobial peptides characterized by having covalently linked N- and C-termini, are promising candidates due to their superior stability and broad-spectrum activity. Here we describe pumicyclin A, a novel circular bacteriocin produced by two nearly identical but independently isolated *Bacillus pumilus* strains. Pumicyclin A exhibits a broad inhibitory spectrum against gram-positive bacteria and is particularly potent against *Listeria* species and *B. cereus*. Genomic analysis revealed a six-gene biosynthetic cluster, with the precursor peptide comprising a 32-amino-acid leader and a 64-amino-acid core, yielding a mature peptide of approximately 6,218 Da. A second downstream gene, encoding a smaller bacteriocin-like peptide, was found to be non-essential for pumicyclin A production. Notably, deletion mutagenesis of the leader peptide to a single methionine residue did not abolish activity, whereas specific substitutions near the circularization site were associated with loss of detectable activity, consistent with impaired biosynthesis (e.g., disrupted circularization, secretion, stability), and indicating key residues important for bacteriocin production. These results offer mechanistic insights into the production of long-leader circular bacteriocins and support pumicyclin A as a promising agent for food biopreservation.

**IMPORTANCE:**

There is an urgent need for safe and effective antimicrobial solutions to control foodborne pathogens and reduce reliance on conventional chemical preservatives. Circular bacteriocins represent a relatively unexplored class of antimicrobials with desirable features, such as high stability and potent antibacterial activity. This study identifies and characterizes pumicyclin A, a new circular bacteriocin from *Bacillus pumilus*, with potent anti-*Listeria* activity and unique biosynthetic characteristics. By dissecting the roles of the leader peptide and conserved residues near the circularization site, we provide novel insights into the structure-function relationships governing circular bacteriocin maturation. These findings lay the groundwork for future (circular) peptide engineering efforts and support the potential application of pumicyclin A as a natural and effective biopreservative to enhance food safety and prolong shelf life.

## INTRODUCTION

Food safety regulations have become a critical global concern, driven by the growing consumer awareness about chemical additives in the products they consume (1, 2). Foodborne pathogens such as *Listeria monocytogenes* and *Bacillus cereus* remain important hazards in food products, although their risk profiles differ substantially: *L. monocytogenes* is of concern even at low levels, whereas *Bacillus cereus* becomes hazardous mainly through growth and toxin production (3, 4). In response to increasing consumer preference for natural preservatives, there is a rising focus on biopreservation, a strategy that harnesses beneficial microorganisms and their metabolites as viable alternatives to synthetic/chemical additives (5). Bacteriocins, especially those produced by generally recognized as safe (GRAS) organisms, have been used for decades as potential food preservatives due to their natural origin and antimicrobial properties (6, 7).

These peptides represent a diverse group of ribosomally synthesized antimicrobials and are produced by many bacterial species (8). They are known for their potent antimicrobial properties and can exhibit narrow-spectrum or broad-spectrum activity (9). This versatility makes bacteriocins valuable agents in a variety of applications. In particular, bacteriocins from GRAS microorganisms hold significant potential (10) as natural food preservatives but also potentially for their applications as therapeutic antimicrobials. Circular bacteriocins represent a subclass within the bacteriocin superfamily, characterized by their head-to-tail ligated peptide backbones that confer enhanced structural stability over linear forms (11). Their robustness, combined with broad antimicrobial activity in many cases, makes them ideal candidates for both food preservation and therapeutic compounds (12).

Most bacteriocins are synthesized as inactive precursor peptides with an N-terminal leader sequence. The leader sequence usually plays a crucial role in mediating bacteriocin maturation processes such as modification and secretion (11). In lantibiotics, for example, leader peptides serve several critical functions (13), including acting as molecular guides to ensure correct post-translational modifications by biosynthetic enzymes, protecting the producer cell by keeping the bacteriocin in an inactive form within the cell, and facilitating the translocation of the bacteriocin to the extracellular environment. However, the role of leader peptides in circular bacteriocins remains poorly understood, particularly due to their high variability in both length and sequence and the absence of conserved motifs, which complicates predictions about their function in maturation (11). Previous studies on circular bacteriocin leader peptides have yielded varying outcomes. For example, truncated leader peptides of enterocin NKR-5-3B completely blocked bacteriocin production (14), whereas for circularin A, bacteriocin production persisted even when the already very small leader was reduced from three amino acids (aa) to a single residue (i.e., methionine) (15).

This study presents the discovery and in-depth characterization of pumicyclin A, a novel circular bacteriocin with a 32-aa leader peptide produced by *Bacillus pumilus. Bacillus* species have a long history of safe use in food fermentations (16), and some, though not all, hold qualified presumption of safety or GRAS status, reflecting their favorable safety profiles (17, 18). Among food-fermenting microorganisms, *Bacillus* spp. are particularly notable for producing a wide spectrum of antimicrobial metabolites (19–22), including bacteriocins, lipopeptides, antibiotics, and other bioactive compounds that contribute to their ecological competitiveness and potential value in food biopreservation (18). Moreover, *Bacillus* spp. are widespread members of the plant microbiome, where they contribute to defense against phytopathogens and herbivores, a role that likely underlies their capacity to produce diverse antimicrobial metabolites (20). This metabolic versatility underscores the relevance of *Bacillus* spp. as a rich source of novel antimicrobial agents.

While several circular bacteriocins have been identified in *Bacillus* (21, 23–27), many remain unexplored (24). Through whole-genome sequencing, we identified the gene cluster responsible for pumicyclin A biosynthesis, together with other potential secondary metabolite biosynthetic gene clusters. Pumicyclin A was purified and heterologously expressed in *Bacillus subtilis* PG10, demonstrating broad-spectrum antimicrobial activity, particularly against the foodborne pathogens *L. monocytogenes* and *B. cereus*. Gene knockouts and peptide mutagenesis, respectively, defined the genetic and aa requirements in the leader sequence and core peptide for bacteriocin production. This work offers insights into the biosynthesis and bioengineering potential of circular bacteriocins, expanding the toolkit for developing novel antimicrobial agents.

## RESULTS

We focused on the identification and characterization of pumicyclin A, a novel circular bacteriocin produced by two closely related *B. pumilus* strains, APC4183 and MG52, with an average nucleotide identity (ANI) score of 96.28%. Although isolated independently in Ireland and the Netherlands, both strains produce pumicyclin A from nearly identical biosynthetic gene clusters.

### Genome analysis and pumicyclin A identification

Draft genomes of *B. pumilus* APC4183 and MG52 were analyzed (Table S1). ANI analysis shows that the genomes share 96.28% identity. Antimicrobial predictions using BAGEL4 and antiSMASH indicated that both strains encode similar secondary metabolite profiles, including fengycin (antifungal lipopeptide), lichenysin (surfactant), and zwittermicin A (a hybrid of a polyketide and a non-ribosomal peptide), as well as several others (Table 1). Notably, both strains encode an identical novel circular bacteriocin, which shares only 39.1% amino acid identity with its closest known homolog, amylocyclicin. This novel bacteriocin is hereafter referred to as pumicyclin A. It is encoded by a distinct six-gene biosynthetic locus and is clearly differentiated from the previously reported *Bacillus pumilus* circular bacteriocins, pumilarin (26) and pumilarin X (21), based on its precursor sequence and predicted physicochemical properties (Table 2). We chose to call it pumicyclin rather than pumilarin to indicate its circular nature, but we do not propose retrospective redesignation of the pumilarins.

**TABLE 1:** Putative secondary metabolites of *Bacillus pumilus* APC4183 and *Bacillus pumilus* MG52 identified by BAGEL4 and antiSMASH^*e*^

Cluster	Secondary metabolites	Putative molecular mass (Da)	Type	Strain	Genome location		Amino acid similarity (%)	Bioactivity	Reference
					Start	Stop			
1	Amylocyclicin	6,381	Bacteriocin^*a*^	APC4183	579,700	589,990	58.04	Antibacterial	(23)
				MG52	152,464	162,754			
2	Bacilysin	270	Other	APC4183	613,796	655,217	85	Antibacterial, antifungal	(28)
3	Bacillibactin	882	NRPS^*b*^	APC4183	882,857	934,583	80	Antibacterial, antifungal	(29)
4	Unknown	–	Betalactone	APC4183	229,080	261,335	–	–	-
				MG52	46,028	78,333			
5	Unknown	–	Type 3 PKS^*c*^	APC4183	714,763	755,863	–	–	–
				MG52	176,184	217,284			
6	Unknown	–	Terpene	APC4183	793,793	815,666	–	–	–
7	Fengycin	1,463.71	Betalactone	APC4183	608,948	637,358	53	Antibacterial, antifungal	(30)
				MG52	459,895	488,312			
8	Lichenysin	1,007–1,035	NRPS	APC4183	179,219	262,921	92	Antibacterial, antifungal	(31)
				MG52	147,160	226,284	85		
9	Zwittermicin A	396.40	NRP + polyketide	APC4183	169,513	250,471	18	Antibacterial	(32)
				MG52	104,140	185,098			
10	Unknown	–	RRE-containing^*d*^	APC4183	5,772	26,677	–	–	–
				MG52	4,318	25,223			
11	Schizokinen	420.41	Terpene, NI-siderophore	APC4183	170,508	208,163	60	Antimicrobial	(33)
				MG52	36,304	73,954			

^*a*^ Bacteriocin is predicted by BAGEL4; other metabolites were predicted by antiSMASH v.7.0.

^*b*^ Non-ribosomal peptide synthase.

^*c*^ Type 3 PKS (polyketide synthase).

^*d*^ The RiPP recognition element (RRE).

^*e*^ "–" indicates not available.

**TABLE 2:** Physiochemical properties of amylocyclicin, amylocyclicin CMW1, pumilarin, and pumicyclin A

Bacteriocin	Length (AA)		α-Helices	MW (Da) (mature)	pI^*a*^	Net charge^*a*^	Hydrophobicity (GRAVY index)^*a*^	Producer	No. of genes	GenBank	Reference
	Leader	Core peptide									
Amylocyclicin	48	64	4	6,381.55	10.41	+5	0.850	*Bacillus amyloliquefaciens* FZB42	6	CP000560.1	(23)
Amylocyclicin CMW1	48	64	4	6,351.59	9.82	+5	0.889	*Bacillus amyloliquefaciens* CMW1	6	DF836084.1	(27)
Pumicyclin A	32	64	4	6,218.30	9.53	+2	1.111	*Bacillus pumilus* APC4183 and MG52	6	JBEPGF000000000.1/QJIZ00000000.1	This work
Pumilarin	38	70	5	7,087.35	10.72	+5	0.579	*B. pumilus* B4107 (re-identified as *B. safensis* B4107)	5	WP_017358050.1 (prepeptide)	(26)

^*a*^ Properties were predicted by the ProtParam tool (https://web.expasy.org/protparam/).

**Fig 1:**
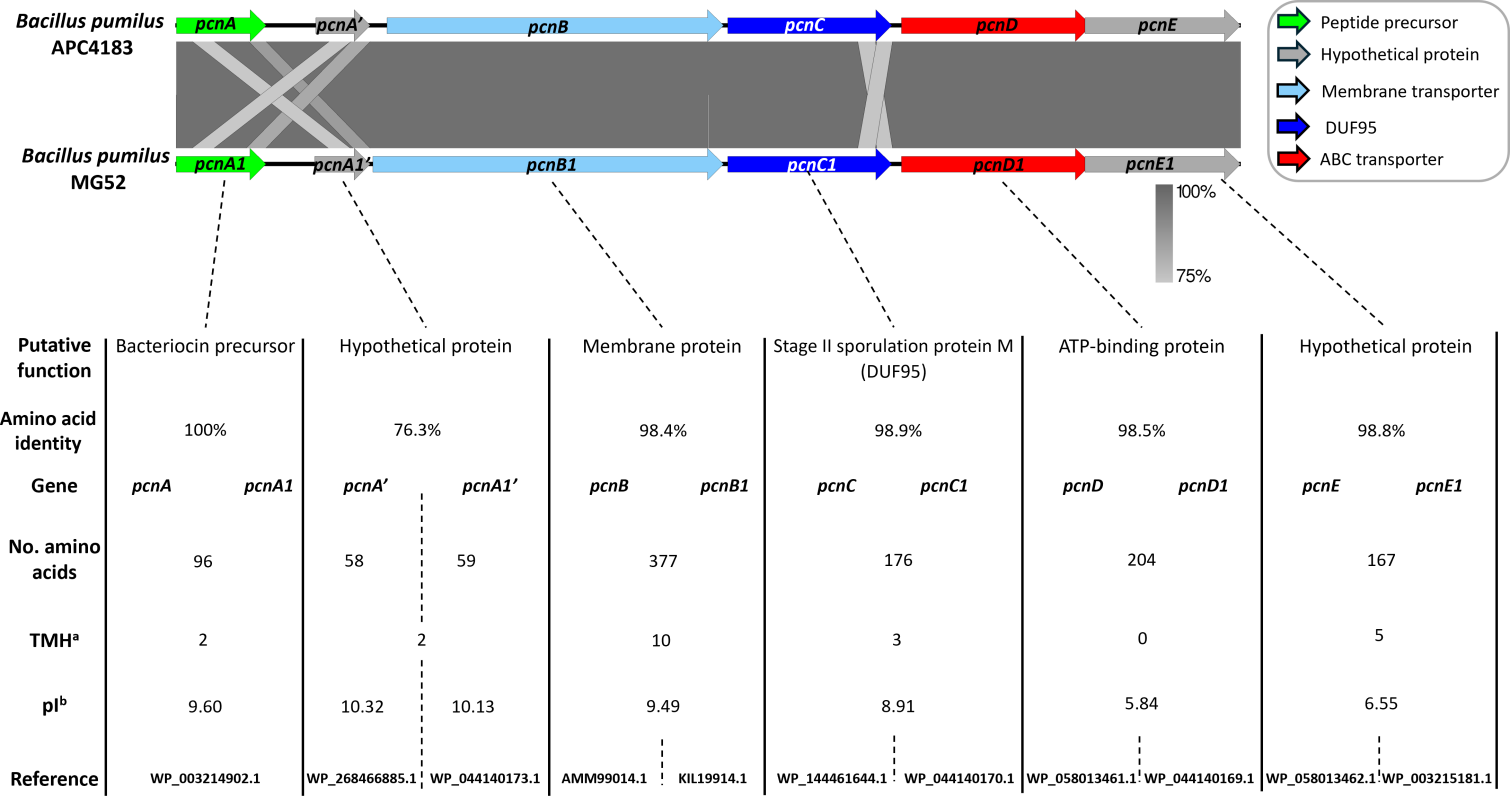
The biosynthetic gene cluster of pumicyclin A and putative characteristics of each gene product encoded by *Bacillus pumilus* APC4183 and *Bacillus pumilus* MG52. ^a^TMH: number of transmembrane helices predicted by the TMHMM online server v.2.0. (https://services.healthtech.dtu.dk/services/TMHMM-2.0/). ^b^pI (theoretical isoelectric point) was predicted with the ExPASy Compute pI/Mw tool (https://web.expasy.org/compute_pi/).

Although both strains potentially produce diverse secondary metabolites, this study primarily focused on pumicyclin A. The biosynthetic gene clusters (Fig. 1) present in both strains share high similarity, with their precursor genes encoding an identical 96-amino-acid peptide. The most significant divergence occurs in the small open reading frames *pcnA′* and *pcnA1′*, located directly downstream of the precursor gene, which encode peptides of 58 and 59 amino acids, respectively. They share only 76.3% amino acid sequence identity, compared with 98%–99% sequence identity for the other four proteins encoded in the cluster. Interestingly, this small peptide also shows high similarity (43.0% identity) to the bacteriocin, even though it is only slightly over half the length of the bacteriocin (Fig. S1).

**Fig 2:**
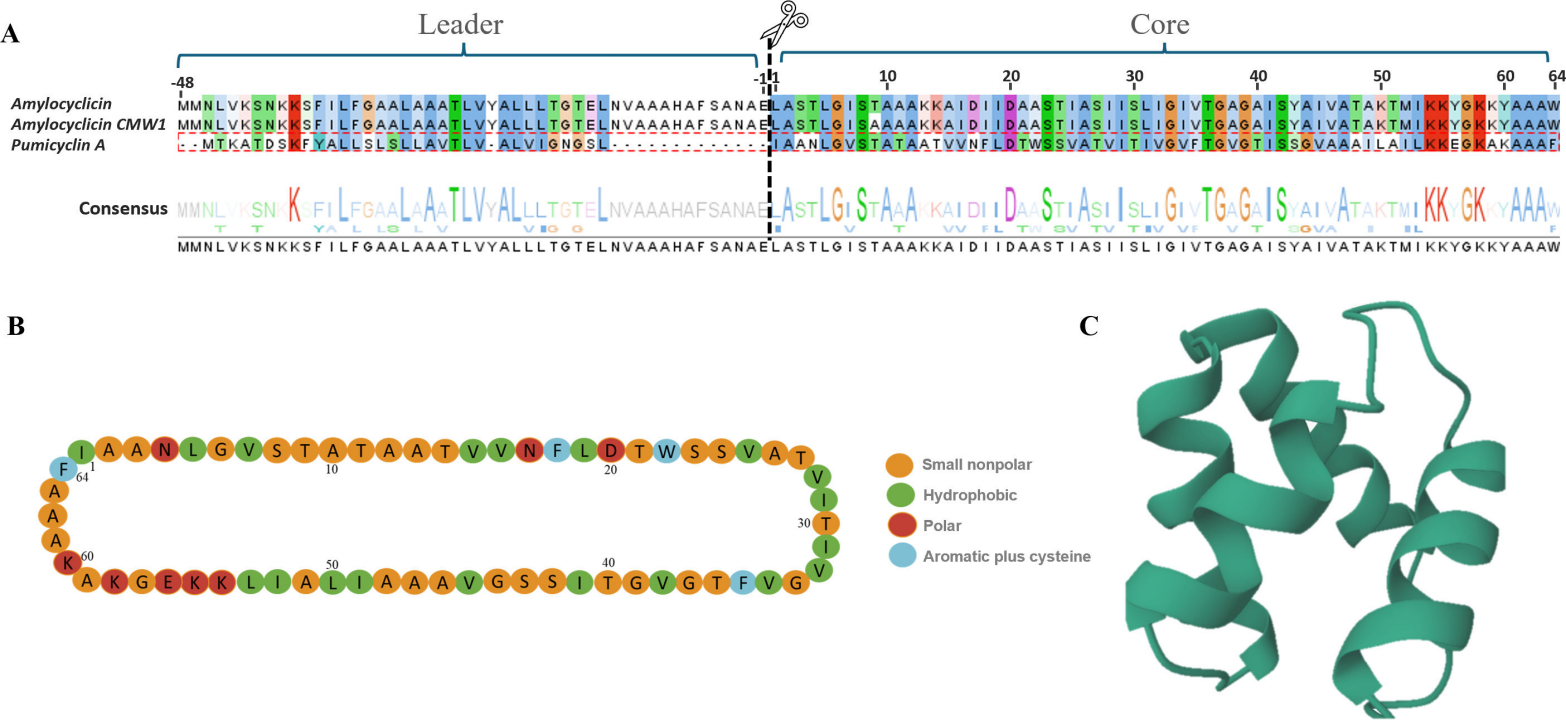
(**A**) MAFFT alignment of pumicyclin A with amylocyclicin and amylocyclicin CMW1; amino acid sequences were colored by Clustal based on their properties and conservation. The consensus sequences are shown in letters. The dotted line and the scissors indicate the predicted cutting site between the leader sequence and the core peptide. (**B**) The predicted circular structure of pumicyclin A, and amino acids are colored by their properties. (**C**) 3D structure predicted by AlphaFold3 server and viewed in Mol* Viewer.

The residue sequence alignment of pumicyclin A with its closest bacteriocin homologs, amylocyclicin and its one-residue variant amylocyclicin CMW1, suggests that its cleavage site lies between leucine and isoleucine, indicating the bacteriocin has a 32-aa leader and a 64-aa core peptide (Fig. 2A). Like many other class I circular bacteriocins (11, 14, 15, 23, 26, 34), the pumicyclin A core peptide begins with a hydrophobic residue (isoleucine) and ends with an aromatic residue (phenylalanine). The predicted 3D model of pumicyclin A (Fig. 2C), generated with AlphaFold3 and visualized in Mol* Viewer, reveals the characteristic saposin-like fold of class I circular bacteriocins with four α-helices: α1 (T9–T21), α2 (V25–T36), α3 (S43–K55), and α4 (K58–L5). Glycine residues (G6, G37, G39, G57) are present within the loops, which could contribute to structural flexibility. Compared to its homologs, amylocyclicin and amylocyclicin CMW1, pumicyclin A has a shorter leader sequence, a lower molecular mass, a lower pI, and a lower net charge but greater hydrophobicity (Table 2).

### Antibacterial activity and production optimization

Antibacterial activity associated with pumicyclin A was assessed using agar-based activity assays with cell-free supernatant generated from the native producer strain *Bacillus pumilus* APC4183 using the freeze-thaw extraction method (freeze-thaw cell-free supernatant [FT-CFS]), a procedure that releases cell-associated compounds and yields a crude preparation. As shown in Table 3, *B. pumilus* APC4183 exhibited a broad-spectrum inhibition against nearly all tested bacterial indicators, including gram-negative strains *Escherichia coli* O156 and *Enterobacter* sp. UCC. In contrast, the FT-CFS exhibited a more selective inhibition profile, targeting only gram-positive bacteria, including *Lactobacillus delbrueckii* subsp. *bulgaricus*, *B. cereus*, and various *Listeria* strains. Notably, this antimicrobial pattern closely resembled that of heterologously expressed pumicyclin A produced in *Bacillus subtilis* PG10 (pMG36c-Pcn), which showed the strongest inhibitory activity against *Listeria* strains, followed by *B. cereus*. In these assays, inhibition zones produced by pMG36c-Pcn were larger than those observed for the native producer. Taken together, these activity comparisons, in conjunction with subsequent matrix-assisted laser desorption/ionization-time of flight (MALDI-TOF) mass spectrometric identification of a peptide matching the predicted pumicyclin A mass following bacteriocin enrichment, are consistent with pumicyclin A being a major contributor to the antibacterial activity detected in the FT-CFS. In addition, the broader inhibition spectrum of the native *B. pumilus* strain suggests the presence of additional antimicrobial metabolites, supported by its genomic potential (Table 1) and the more limited activity of pumicyclin A alone. Due to the pronounced anti-*Listeria* activity of pumicyclin A, *Listeria innocua* was selected as the indicator strain to evaluate the antibacterial efficacy of pumicyclin A from the FT-CFS fraction during production optimization.

**TABLE 3:** Antibacterial activity of *Bacillus pumilus* APC4183, its FT-CFS, and heterologously produced pumicyclin A (*B. subtilis* PG10, pMG36c-Pcn) assessed by colony overlay and well-diffusion assays

Indicator	Source^*b*^	Inhibition zone (mm)^*a*^		
		Colony overlay assay (native producer APC4183)	Well-diffusion assay (FT-CFS)	Colony overlay assay (heterologous expression; PG10, pMG36c-Pcn)
Gram-positive strains				
*Listeria innocua*	UCC	6.00 ± 0.02	6.35 ± 0.01	15.33 ± 0.66
*Listeria monocytogenes*	EDGe	3.97 ± 0.03	5.29 ± 0.53	17.23 ± 0.25
*Bacillus cereus*	DPC 6087	8.57 ± 0.94	3.12 ± 0.23	12.80 ± 0.75
*Lactobacillus delbrueckii* subsp. *bulgaricus*	LMG 6901	23.61 ± 0.22	6.27 ± 0.18	ND
*Leuconostoc mesenteroides*	APC4234	2.58 ± 0.17	−	9.00 ± 1.00
*Clostridium perfringens*	EM124	28.57 ± 0.06	−	+/−
*Staphylococcus aureus*	DPC 5645	19.82 ± 1.38	−	−
*Staphylococcus epidermidis*	DSM 3095	23.43 ± 1.52	−	−
Gram-negative strains				
*Escherichia coli*	DPC 6054	5.25 ± 0.69	−	−
*Enterobacter cloacae*	UCC	3.91 ± 0.18	−	ND
*Pseudomonas aeruginosa*	APC2064	−	−	−

^*a*^ Values are expressed as average ± standard deviation (*n* = 3). –, inactive; ND, test not done.

^*b*^ All indicators are collected from APC Microbiome Ireland, University College Cork (UCC).

To assess conditions that enhance anti-*Listeria* activity, we evaluated the culture conditions for *B. pumilus* APC4183 by testing various carbon and nitrogen sources. The growth medium was altered by using various carbon (30 g/L) and nitrogen (15 g/L) sources. During the assay, APC4183 exhibited reduced growth when glycerol was supplied as the carbon source, whereas growth was comparable across the other carbon sources and all nitrogen sources tested. Since FT-CFS fractions were prepared directly from agar plates using the freeze-thaw method and bacterial biomass was not quantified, anti-*Listeria* activity was not normalized to cell density. Consequently, the observed activity reflects the combined effects of bacterial growth and anti-*Listeria* production under the conditions tested. Among the conditions examined, supplementation with sucrose as the carbon source and meat extract as the nitrogen source resulted in the highest observed anti-*Listeria* activity (Fig. S2). Using this growth condition, the FT-CFS fraction containing pumicyclin A was prepared and tested for antibacterial activity against a broad panel of *Listeria* species, including *L. ivanovii*, *L. grayi,* and 10 *L. monocytogenes* isolates (Table 4). The results showed potent inhibitory activity against nearly all tested strains, with only *L. monocytogenes* 33068 showing no detectable inhibition. This strain-specific insensitivity may provide an opportunity for future studies aimed at probing the mode of action of pumicyclin A.

**TABLE 4:** Antimicrobial activity of pumicyclin A against *Listeria* strains using FT-CFS

*Listeria* species	Inhibition zone (mm)^*a*^
*Listeria ivanovii* 293	9.37 ± 0.38
*Listeria grayi* 671	5.46 ± 0.12
*Listeria monocytogenes* 33068	–^*b*^
*Listeria monocytogenes* 33186	2.19 ± 0.01
*Listeria monocytogenes* 33077	2.33 ± 0.03
*Listeria monocytogenes* F5817	2.43 ± 0.16
*Listeria monocytogenes* 33093	2.87 ± 0.53
*Listeria monocytogenes* 33038	3.11 ± 0.01
*Listeria monocytogenes* 33176	6.01 ± 0.09
*Listeria monocytogenes* 33225	6.06 ± 0.05
*Listeria monocytogenes* 33013	6.14 ± 0.29
*Listeria monocytogenes* J1-220	7.10 ± 0.15

^*a*^ Values are expressed as average ± standard deviation (*n* = 3).

^*b*^ "–" indicates inactive.

### Characterization, purification, and MALDI-TOF analysis

To fractionate and enrich the antibacterial compound, the FT-CFS fraction was subjected to two independent methods: Amicon centrifugal filtration with a 10 kDa molecular weight cut-off and solid-phase extraction using a Strata C18-E column. Following Amicon filtration, antibacterial activity was detected exclusively in the >10 kDa retentate, with no activity observed in the corresponding filtrate (Table S2a). This result suggests that the active compound is either larger than 10 kDa or forms aggregates. In parallel, C18 SPE analysis showed that the active compound eluted in isopropanol (IPA) concentrations between 40% and 80%.

**Fig 3:**
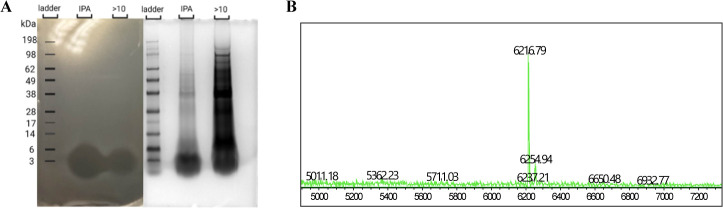
(**A**) SDS-PAGE analysis of the >10 kDa and IPA fractions: left, gel overlaid with *L. innocua* UCC showing inhibition zones; right, corresponding stained gel with active fractions. (**B**) MALDI-TOF mass spectrum of the 70% isopropanol elute, showing the molecular mass of pumicyclin A (theoretical mass: ~6,218 Da).

Active fractions from both the >10 kDa retentate and the 70% IPA eluate were subsequently analyzed by gradient SDS-PAGE. Two parallel lanes were run: one stained for protein visualization and the other overlaid with *Listeria innocua* to localize antibacterial activity (Fig. 3A). In both cases, activity localized to a peptide band migrating at an estimated molecular weight of 3–6 kDa. MALDI-TOF mass spectrometric analysis of the 70% IPA eluate revealed a dominant peak at 6,216.79 (±2) Da (Fig. 3B), corresponding closely to the predicted mass of mature pumicyclin A (6,218.3 Da). This observation supports the presence of pumicyclin A in the enriched fraction of the FT-CFS and is consistent with the antibacterial activity profile observed for heterologously produced pumicyclin A. Interestingly, pumicyclin A was initially retained in the >10 kDa retentate despite its predicted molecular mass of 6,218.3 Da, suggesting that its hydrophobic nature may promote aggregation, preventing it from passing through the 10 kDa filter. To test this, FT-CFS was treated with increasing concentrations of methanol (0%, 25%, 50%, 75%) to disrupt hydrophobic interactions. As shown in Table S2b, anti-*Listeria* activity progressively shifted to the <10 kDa fraction at higher methanol concentrations, supporting the hypothesis that pumicyclin A tends to aggregate under native conditions.

### Heterologous expression of pumicyclin A in *Bacillus subtilis* PG10

**Fig 4:**
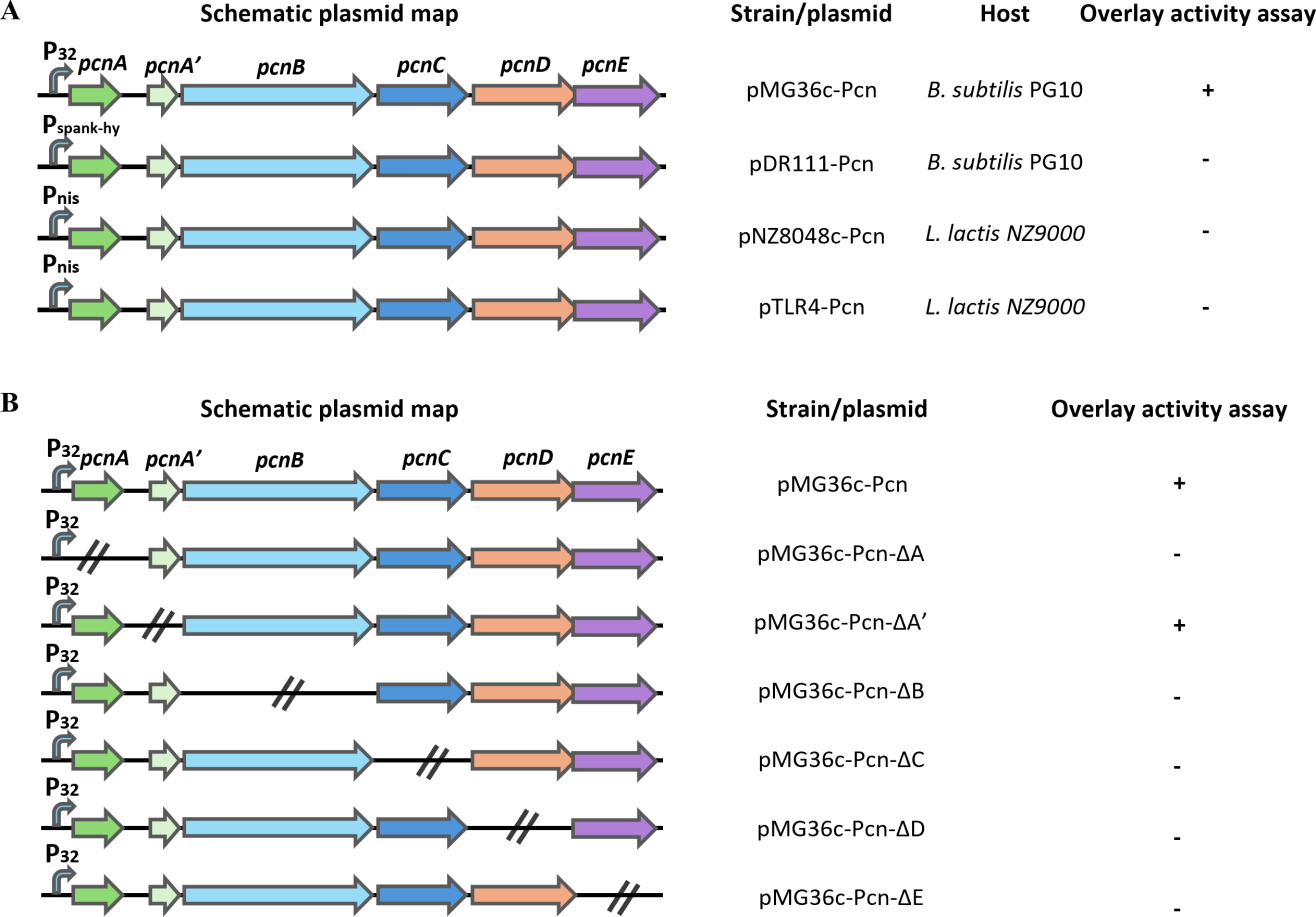
Identification of genes responsible for the production and maturation of pumicyclin A. (**A**) Schematic representation of constructed plasmids and their productivity of the pumicyclin A. (**B**) Schematic representation of gene knockouts using the plasmid pMG36c with a constitutive promoter p32. Indicator strain: *Micrococcus flavus* NIZO B423.

The genome-minimized strain *B. subtilis* PG10 (35, 36) was used for the heterologous production of pumicyclin A. Two plasmids were tested: pMG36c, a broad host-range plasmid, and pDR111, which can integrate into the *Bacillus* genome. Overlay activity assays showed that the pMG36c construct successfully produced clear inhibition zones against the tested indicator strain *Micrococcus flavus*, confirming effective pumicyclin A expression in *B. subtilis* PG10 (Fig. 4A). However, no inhibition zones were observed when pumicyclin A was expressed under the nisin-inducible system in *Lactococcus lactis*, although trace amounts of bacteriocin were detected after purification (data not shown). It is plausible that a similar outcome occurred when pumicyclin A was expressed from the integrated plasmid pDR111 in *B. subtilis* PG10. This indicated that genomic integration resulted in substantially lower transcription levels of the bacteriocin genes relative to the pMG36c construct, thereby limiting pumicyclin A production to levels insufficient for detection in overlay assays. Therefore, pMG36c-based constructs in *Bacillus subtilis* PG10 were used for all subsequent mutagenesis and functional analyses. The expression vectors and host systems evaluated in this study are summarized in Table 5.

**TABLE 5:** Summary of heterologous expression vectors and host systems evaluated for pumicyclin A production

Vector	Type	Host	Overlay activity	Experimental application
pMG36c (37)	Broad-host-range plasmid, constitutive P32 promoter	*B. subtilis* PG10	Robust activity (primary system)	Functional overlays, antimicrobial spectrum assays, mutagenesis
pDR111 (38)	Chromosomal integration vector, IPTG-inducible hyper-spank promoter (P_spank-hy_)	*B. subtilis* PG10	No detectable activity	Expression analysis
pNZ8048c (39)	High-copy plasmid with NICE system (nisin-inducible production)	*L. lactis* NZ9000	No detectable activity	Expression analysis
pTLR4 (40)	Low- to medium-copy plasmid with NICE system	*L. lactis* NZ9000	No detectable activity	Expression analysis

**Fig 5:**
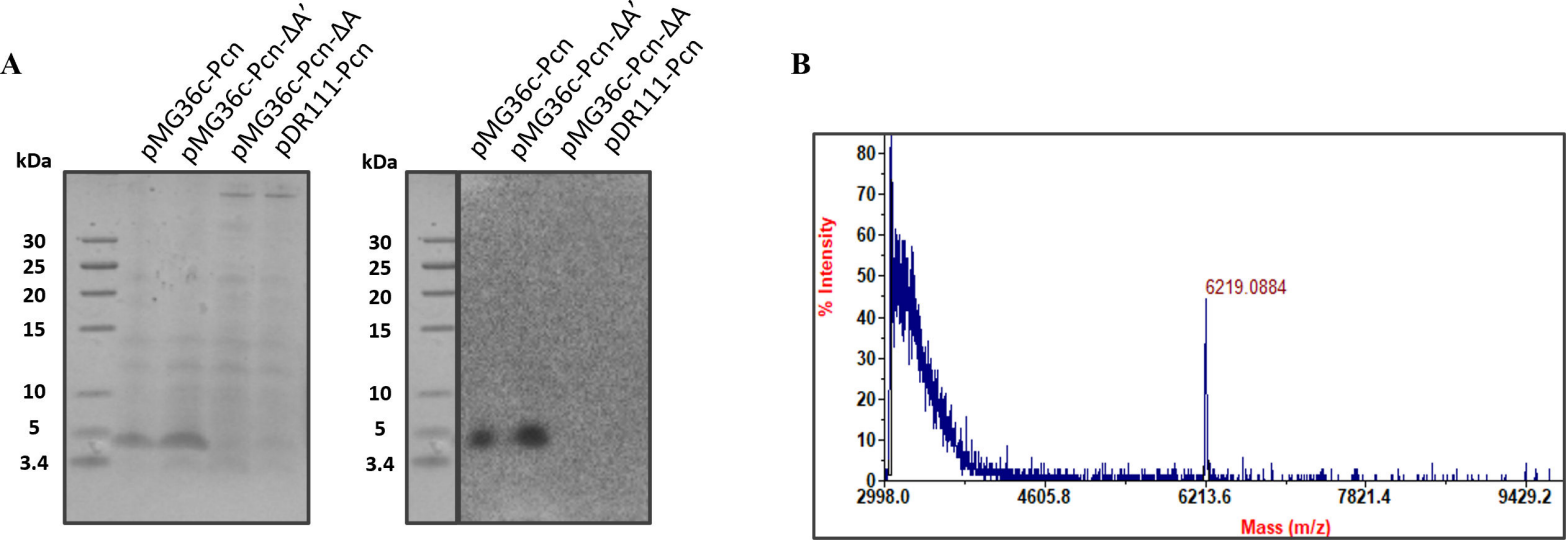
Peptide production and MS confirmation of pumicyclin A. (**A**) Peptide production (left) and antimicrobial activity (right) of four expression constructs using tricine-SDS-PAGE protein gel analysis. Indicator strain: *Micrococcus flavus* NIZO B423. (**B**) MALDI-TOF spectrum for molecular mass detection to confirm the production and modification of pumicyclin A (theoretical mass: ~6,218 Da).

As previously mentioned, we identified a gene encoding a second small bacteriocin-like peptide within the pumicyclin A gene cluster, a feature rarely observed in other characterized circular bacteriocin gene clusters. Although the peptide is only slightly more than half the length of the predicted bacteriocin, it still shares high sequence similarity across the conserved core region (Fig. S1). To investigate the role of this peptide in antimicrobial production and to assess the functionality of other biosynthetic genes, we performed gene-knockout scans of the six-gene cluster (Fig. 4B). The impact of each gene knockout was first evaluated using overlay activity assays. The results indicated that the small bacteriocin-like peptide is not essential for bacteriocin production, while the other five genes appear to be required for bacteriocin maturation and/or secretion. The heterologously produced active peptides were further analyzed by tricine-SDS-PAGE and MALDI-TOF to confirm correct modification (Fig. 5).

### Site-directed leader mutagenesis and leader truncations

The bacteriocin structural gene encodes a 32-aa leader and a 64-aa core peptide. The maturation process for circular bacteriocins requires leader cleavage and circularization. To investigate the role of this long leader in bacteriocin maturation, we conducted site-directed mutagenesis (Table 6), including single-residue mutations, multiple-residue mutations, and His-tag addition.

**TABLE 6:** Overview of the mutant variants generated from site-directed mutagenesis and their effects on antimicrobial activity

Leader mutants	Activity^*a*^	I1 mutants	Activity	A2 mutants	Activity	A63 mutants	Activity	F64 mutants	Activity
L-1A	+	I1L	+	A2D	−	A63D	−	F64A	+
L-1D	+	I1V	+	A2I	−	A63I	−	F64W	+
L-1K	+	I1M	+	A2K	−	A63K	−	F64Y	+
K-30A	+	I1A	−	A2L	−	A63L	−		
D-27A	+			A2V	+	A63V	−		
K-25A	+								
K-25A/F-24A/Y-23A	+								

^*a*^ Activity: “+” indicates active; “−” indicates inactive.

For single-residue mutations in the leader peptide, we focused on charged residues (K-30, D-27, K-25) and the leucine residue (L-1) at the leader-cleavage site. We substituted the charged residues with alanine and the leucine residue with either alanine, negatively charged aspartate, or positively charged lysine, resulting in six mutants: K-30A, D-27A, K-25A, L-1A, L-1D, and L-1K. Given that aromatic residues are often present in leaders of circular bacteriocins and can significantly influence bacteriocin production (15), we also created a mutant with three consecutive residues altered, resulting in the mutant K-25A/F-24A/Y-23A. Additionally, we examined the impact of adding a His-tag to the leader on bacteriocin production efficiency. The overlay activity tests demonstrated that all tested site-directed leader mutants maintained bacteriocin production and produced antimicrobial inhibition zones. Interestingly, the addition of a His-tag notably enhanced bacteriocin production efficiency, resulting in significantly larger inhibition halos than those of other substituted mutants and even surpassing those of the wild type (Fig. S3).

**Fig 6:**
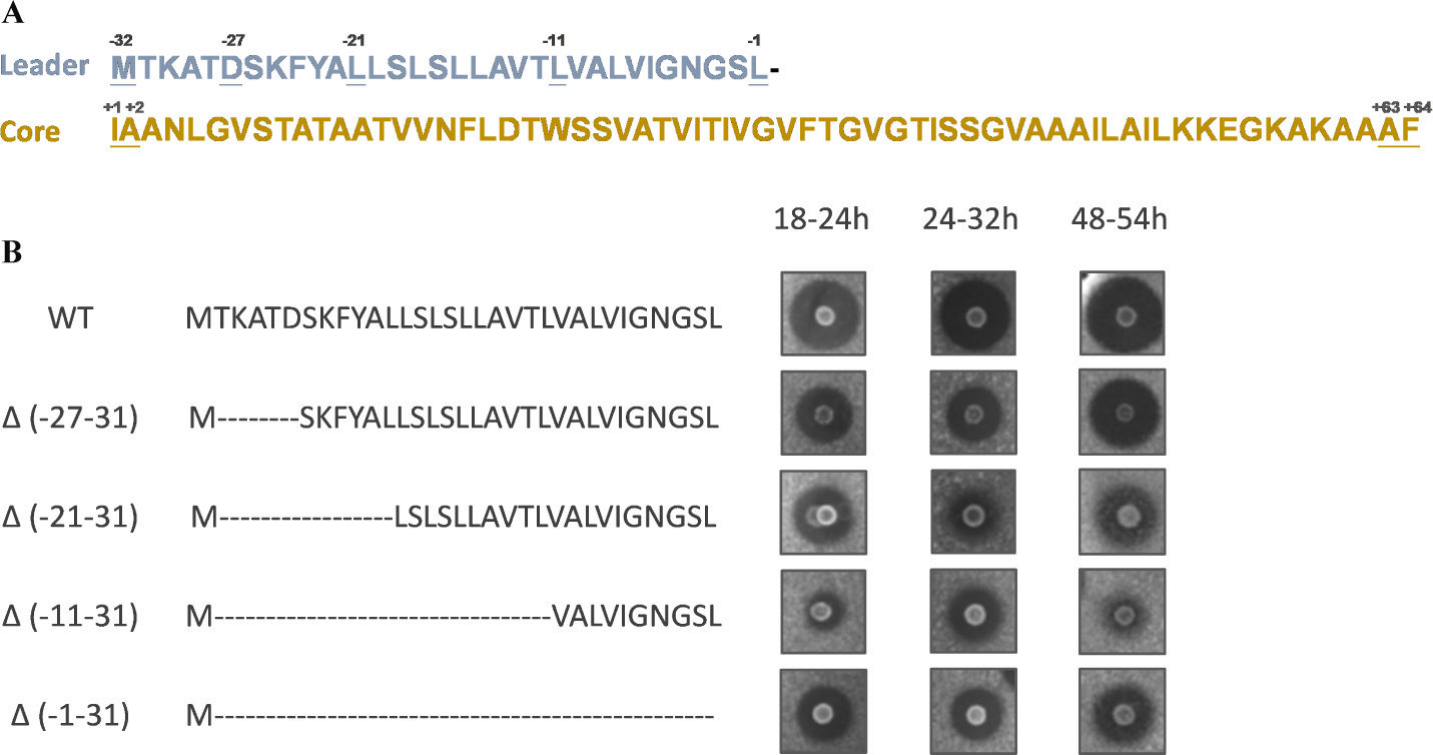
Effect of leader truncations on pumicyclin A production. (**A**) Amino acid sequence of the precursor peptide of pumicyclin A. (**B**) Impact of leader truncations on bacteriocin production at various incubation times. Indicator strain: *Micrococcus flavus* NIZO B423.

To delve deeper into this aspect, we conducted leader truncation assays (Fig. 6). Truncating the leader to varying lengths revealed a general trend: shorter leaders corresponded to decreased antimicrobial production, consistent with the effects observed with His-tag addition, which boosted bacteriocin production. However, intriguingly, the construct with the shortest possible leader, a single methionine, produced inhibition zones larger than those with an 11-aa leader. Colony mass spectrometry (21), performed directly on colonies without peptide purification, indicated that the Δ(-21–31) construct (21-aa leader) yielded the clearest mature bacteriocin signal, while both longer and shorter leaders produced less distinct spectra with slight mass deviations (Fig. S4). Moreover, prolonging the incubation time of the producing culture from 18 h to 48 h did not diminish inhibition zones, indicating the stability of the bacteriocin.

### Site-directed mutagenesis of residues near the circularization site of the core peptide

Circular bacteriocins are characterized by head-to-tail ligation, connecting the N- and C-terminus of the core peptides, and the ligation site is located within a helical structure surrounded by hydrophobic regions (11). In this study, we performed site-directed mutagenesis on four residues near the ligation site: the first two residues, I1 and A2, and the last two residues, A63 and F64, of the core peptide. Table 6 provides an overview of the mutant variants generated in this study and their effects on antimicrobial activity.

Previous studies on circularin A, a circular bacteriocin with a short leader of three amino acids, have shown a preference for hydrophobic residues with short side chains at the first two positions (+1 and +2) (15). Our findings indicate that pumicyclin A can accommodate bulky hydrophobic residues at position +1 while retaining activity; however, substitution with a smaller residue, specifically alanine, failed to produce an active peptide. At position +2, smaller hydrophobic residues are required, similar to circularin A. Unlike circularin A, which accepts only aromatic residues at the last position and any hydrophobic residues at the second-to-last position, pumicyclin A allows both aromatic residues and alanine at the last position but is extremely limited/selective about residues at the second-to-last position, failing to generate an active peptide for all tested variants when substituted with either hydrophobic or charged residues. Notably, circularin A belongs to the short leader sequence group, whereas pumicyclin A belongs to the long leader sequence group. The potential differences in their biosynthetic mechanisms remain unclear.

### Antimicrobial spectrum of pumicyclin A

The antimicrobial spectrum of pumicyclin A was evaluated using an overlay activity assay against a panel of gram-positive and gram-negative bacteria, including foodborne pathogens and clinically relevant multidrug-resistant strains such as *L. monocytogenes*, *B. cereus*, and *Staphylococcus aureus* (Table 7). The results demonstrated that pumicyclin A is a broad-spectrum bacteriocin effective against a wide range of gram-positive bacteria, but not gram-negative bacteria. Notably, pumicyclin A exhibited significant activity against foodborne pathogens like *L. monocytogenes* and *B. cereus*. It was also active against many other gram-positive bacteria, including *Leuconostoc mesenteroides* and *Clostridium perfringens* (Table 7). In contrast, all tested gram-negative bacteria, such as *E. coli*, *P. aeruginosa*, *Cronobacter sakazakii*, and *Klebsiella pneumoniae*, were insensitive to pumicyclin A.

**TABLE 7:** Antimicrobial spectrum of pumicyclin A, assessed via overlay assays using the heterologous producer *B. subtilis* PG10 (pMG36c-Pcn)

Indicators	Code	Inhibition zone (mm)^*a*^
Gram-positive strains		
*Listeria monocytogenes*	EDGe	+++
*Listeria innocua*	UCC	+++
*Listeria ivanovii*	293	+++
*Listeria grayi*	671	+++
*Bacillus cereus*	DPC 6087	++
*Bacillus subtilis*	APC4236	−
*Bacillus pumilus*	APC4237	−
*Bacillus australimaris*	APC4239	−
*Bacillus wiedmannii*	APC4238	−
*Limosilactobacillus fermentum*	APC249	++
*Lentilactobacillus buchneri*	APC240	−
*Lactiplantibacillus plantarum*	APC4235	−
*Latilactobacillus sakei*	APC243	−
*Staphylococcus aureus*	DPC 5645	−
*Staphylococcus capitis*	APC2923	−
*Staphylococcus epidermidis*	DSM 3095	−
*Staphylococcus pseudointermedius*	DK 279	+
*Micrococcus luteus*	APC4061	+++
*Micrococcus flavus*	NIZO B423	+++
*Leuconostoc mesenteroides*	APC4234	+
*Lactococcus lactis*	HP	++
*Clostridium perfringens*	EM124	+/−
*Enterococcus faecalis*	ATCC 29200	−
*Enterococcus faecium*	DPC 3675	−
*Enterococcus* sp. (VRE)	APC1026	−
*Enterococcus* sp. (VRE)	APC1028	−
*Streptococcus agalactiae*	35	−
Gram-negative strains		
*E. coli*	DPC 6054	−
*P. aeruginosa*	PA01	−
*C. sakazakii*	DPC 6440	−
*K. pneumoniae*	NCIMB 13218	−

^*a*^ 0 mm–10 mm (+), 10 mm–15 mm (++), >15 mm (+++), inactive (−). VRE, vancomycin-resistant *Enterococcus*.

## DISCUSSION

This manuscript describes the identification of a new circular bacteriocin in two independently isolated *B. pumilus* strains, APC4183 and MG52, which share a 96.28% ANI across their genomes. Draft genomes of these strains revealed similar profiles of gene clusters encoding antimicrobial peptides and secondary metabolites, including a novel circular bacteriocin we named pumicyclin A, which has a predicted molecular mass of 6,218 Da. In this study, we performed a detailed investigation into this bacteriocin in terms of its antimicrobial spectrum, production and purification, and by cloning its biosynthetic gene cluster into a heterologous host, identified the requisite genes and some of the amino acids within the antimicrobial peptide required for antimicrobial activity. The identification of the same bacteriocin in two geographically distinct *B. pumilus* strains points to a conserved competitive role within the species. BLAST analysis further reveals that pumicyclin A has close homologs across several *Bacillus* species and even related genera (Table S3), highlighting the wider distribution of this circular bacteriocin family.

Genome analysis indicated that pumicyclin A is encoded within a six-gene cluster in both strains APC4183 and MG52 (Fig. 1). Although both strains share an identical bacteriocin sequence, the amino acid sequences of their biosynthetic proteins differ slightly, with PcnA′ and PcnA1′ showing the least similarity (76.3% identity), while the remaining four genes share over 98% similarity. This gene cluster is responsible for bacteriocin maturation and secretion and includes genes encoding the precursor peptide, a large membrane protein, the DUF95 protein characteristic of circular bacteriocins, and an ATP-binding protein (all key components typically found in circular bacteriocin synthesis), as well as two hypothetical proteins. Knockout assays of a heterologously expressed *pcnA1A1′B1C1D1E1* cluster showed that among the six genes, only *pcnA1′* is non-essential for bacteriocin maturation and secretion, despite its high similarity to the precursor peptide (Fig. S1). This gene encodes a small cationic peptide with two transmembrane domains, characteristics typical of many other self-immunity proteins in circular bacteriocins, such as CirE and As-48D1, suggesting that PcnA1′ possibly functions as an immunity protein. Similar findings were observed in the amylocyclicin gene cluster, where the knockout of the *acnF* gene (encoding an immunity protein) did not disable bacteriocin production (23). Additionally, the other hypothetical protein, PcnE1, is predicted to be an additional membrane protein with five transmembrane domains—an element not consistently found in circular bacteriocin clusters, similar to As-48C1 in the AS-48 cluster (41). Knockout assays have also shown that *pcnE1* is essential for bacteriocin biosynthesis, consistent with findings for *as-48C1*.

**Fig 7:**
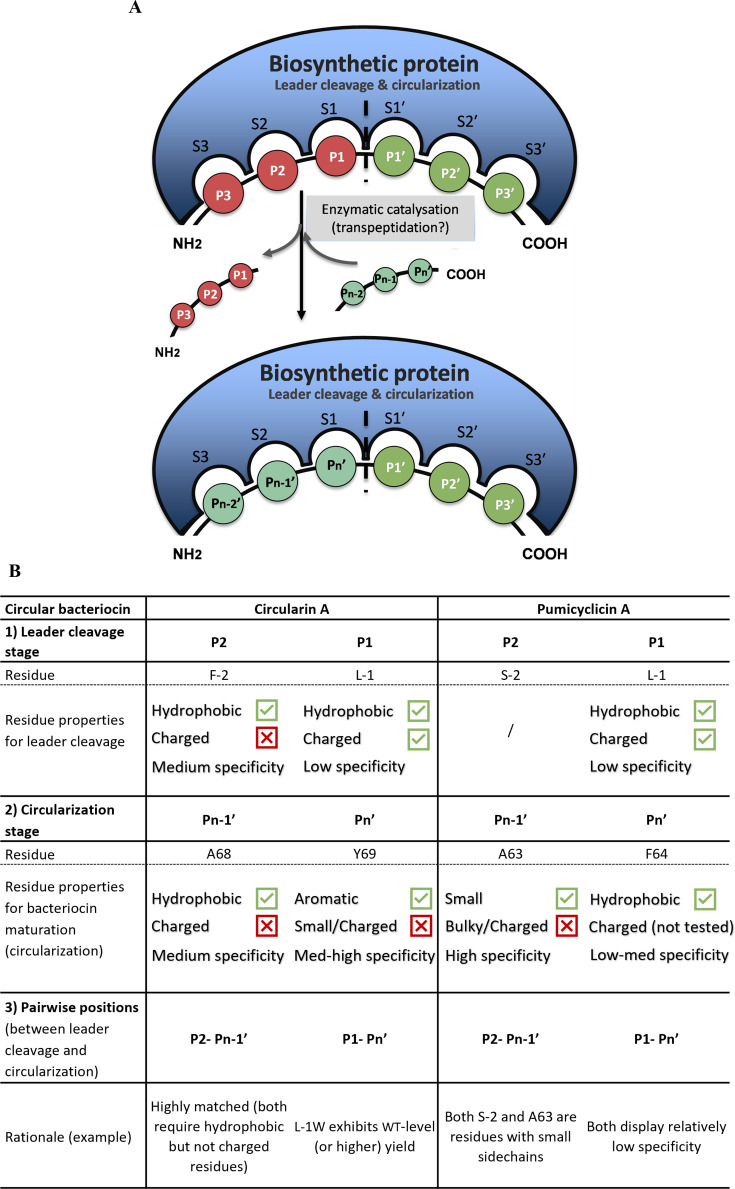
Mechanism proposed for a single enzyme mediating both leader peptide removal and circularization of circular bacteriocins. (**A**) Schechter-Berger nomenclature. The amino acid residues of the substrate, designated P1, P2, P3, etc., interact with the corresponding enzyme-binding pockets, S1, S2, S3, etc. residues and pockets located C-terminal to the cleavage site are denoted P1′ and S1′, respectively. (**B**) Residue properties required for leader cleavage and circularization, as determined by mutagenesis studies. Size and charge preferences were primarily investigated: to assess charge effects, charged and hydrophobic residues were tested; to assess size preferences, hydrophobic residues with small or large side chains were examined. *n* = the number of amino acids in the corresponding mature bacteriocin (i.e., *n* = 69 for circularin A and *n* = 64 for pumicyclin A).

The leader peptide is generally thought to be required to direct post-translational modifications essential for bacteriocin maturation (11, 42). The role of leader peptides in the biosynthesis of many bacteriocins such as lantibiotics is well understood, where leader peptides interact with biosynthetic enzymes via specific residues (e.g., the FNLD box in nisin A) (43, 44). However, the function of leaders in circular bacteriocins remains unclear (11). The considerable variation in sequence and length among these leader peptides complicates predictions about their role in bacteriocin biosynthesis. Leader truncation studies have been used to explore circular bacteriocins with both short and long leader sequences, such as circularin A (15) and enterocin NKR-5-3B (14), respectively. For circularin A, the leader could be truncated to a single residue without disrupting bacteriocin maturation (15), whereas any truncation of the enterocin NKR-5-3B leader completely prevents its production (14). In this study, we further demonstrate that a one-residue leader (specifically, methionine) can still result in the production of a mature and active circular bacteriocin (Fig. S5), even for a bacteriocin that naturally has a long leader sequence. These findings suggest that the key sequence(s) recognized by biosynthetic proteins for correct modifications primarily reside in the core peptide region rather than in the leader peptide itself. While circular bacteriocins with either short or long original leaders can be successfully produced using modified constructs with a single methionine residue as the leader, substitutions in the core peptide near the ligation site revealed different patterns. The primary differences were observed in the first and second-to-last residues (positions +1 and +63). Compared to circularin A, pumicyclin A could accommodate larger residues at position +1 but required smaller residues at position +63. This may reflect the size-dependent constraints of the catalytic pocket in the biosynthetic enzyme(s) that is involved in leader cleavage and circularization of circular bacteriocins (Fig. 7). Notably, the residue requirements at the second-to-last position Pn-1′ of the core peptide closely reflect the P2 residue of the leader peptide in both circularin A and pumicyclin A (i.e., a bulky F−2 in circularin A and a small S−2 in pumicyclin A). This trend appears to represent a generalized pattern, in which long-leader circular bacteriocins preferentially contain a small residue at position −2, whereas short-leader circular bacteriocins tend to harbor a relatively bulkier residue at this position (Fig. S6). Consistent with this pattern, unlike the A68 substitutions tolerated in circularin A, replacing A63 with bulky residues in pumicyclin A abolished production of an active peptide (Table 6).

Based on systematic mutagenesis of residues at both the leader cleavage site and the ligation site in circularin A and pumicyclin A, we propose that a single enzyme mediates both leader peptide removal and circularization. In this model, the P2–Pn-1′ residue pairs are primarily influenced by the size and charge constraints of the catalytic pocket, whereas the P1–Pn′ pairs are further constrained by the requirements of the ligation reaction, creating greater variation in the P1–Pn′ pairs than in the P2–Pn-1′ pairs (Fig. 7). Circular bacteriocins typically adopt a compact structure comprising four to five α-helices, with the ligation site embedded within a helical region (11). Our results show that substitution of residues at the ligation site with charged amino acids is unfavorable for bacteriocin production, whereas charged residues are tolerated at the −1 position of the leader peptide, consistent with the frequent presence of charged amino acids at this position in native circular bacteriocin leaders (Fig. S6). This observation indicates that residues at the ligation site are subject to more stringent sequence constraints than those at the cleavage site. Consequently, residues within the leader peptide generally exhibit lower specificity than their counterparts in the core peptide. Given that DUF95-containing proteins have been implicated in leader peptide cleavage (45), we propose a model in which the DUF95 protein mediates both leader peptide cleavage and circularization (Fig. 7). Notably, head-to-tail cyclized peptides are not unique to bacteria but also occur in eukaryotes, such as plant cyclotides, which are ~30 aa in length and are biosynthesized (cyclized) by cysteine proteases (asparaginyl endopeptidases) via transpeptidation reactions (46). Unlike cyclotides, circular bacteriocins lack disulfide bonds and C-terminal extensions, and the enzymatic mechanism by which their “free” C-terminal end is activated for nucleophilic attack by the N-terminal amino group remains unresolved (11).

The antimicrobial activity spectra of reported circular bacteriocins vary significantly, including broad-spectrum bacteriocins like enterocin AS-48 (47) and narrow-spectrum bacteriocins such as plantaricyclin A (48). The circular bacteriocin identified in this study, pumicyclin A, is a broad-spectrum bacteriocin active against a wide range of gram-positive bacteria, though it does not affect gram-negative bacteria. Notably, pumicyclin A demonstrated potent antimicrobial activity against *L. monocytogenes*, a significant foodborne pathogen that can contaminate food during processing and cause severe infections in immunocompromised individuals (49), and *B. cereus*, a major foodborne pathogen responsible for emetic and diarrheal foodborne illnesses (50). This highlights the potential of pumicyclin A to reduce contamination risks posed by *L. monocytogenes* and *B. cereus* in food systems. Nevertheless, further studies are required to evaluate its efficacy and stability in real food matrices, particularly in comparison with established bacteriocins such as nisin, the first FDA-approved bacteriocin for food use (51, 52). Notably, the producer strain of carnocyclin A, *Carnobacterium maltaromaticum* UAL307, is already approved for food preservation in the United States and Canada (53), underscoring the strong feasibility of employing circular bacteriocins for biopreservation.

In conclusion, pumicyclin A is a novel circular bacteriocin identified in two *Bacillus pumilus* isolates that represents a promising antimicrobial agent with broad-spectrum activity against gram-positive bacteria, particularly against foodborne pathogens such as *L. monocytogenes* and *B. cereus*. The successful heterologous production of pumicyclin A with only a one-residue leader suggests that its maturation is primarily directed by the core peptide sequence, particularly residues near the ligation site that form a *motif* favorable for bacteriocin maturation. This contrasts with many other bacteriocin types, where the leader peptide is critical for proper maturation. Although a leader peptide is not strictly required for circular bacteriocin production, when present, the residue requirements at the penultimate position (Pn−1′) of the core peptide closely reflect the P2 residue of the leader peptide. This observation strongly suggests that a single enzyme mediates both leader peptide removal and circularization. Overall, these findings not only advance understanding of circular bacteriocin biosynthesis but also underscore the potential of pumicyclin A for food safety applications. By targeting critical pathogens in food systems, pumicyclin A holds promise as a natural preservative to mitigate contamination risks posed by *L. monocytogenes* and *B. cereus*, supporting its further exploration as an effective biopreservative.

## MATERIALS AND METHODS

### Bacterial strain isolation and genome analysis

Two soil-derived *B. pumilus* strains, APC4183 and MG52, were independently isolated from Cork, Ireland, and the Netherlands, respectively. Both strains were selected for their potent antimicrobial activity against various gram-positive bacteria and their shared ability to produce the same novel circular bacteriocin. Both strains have been genome sequenced: the sequence of strain MG52 was obtained as previously described (54); genomic DNA of strain APC4183 was extracted using the GenElute Bacterial Genomic DNA Kit and quantified with a Qubit 2.0 Fluorometer. Whole-genome sequencing was performed on the Illumina MiSeq platform (2 × 250 bp paired-end reads) by MicrobesNG. Genome assembly quality, completeness, and contamination were evaluated using QUAST v.5.0.2 and CheckM v.1.0.18.

Annotation was conducted using Prokka v.1.14.6 via the European Galaxy server (usegalaxy.eu). The ANI between the two strains was calculated using FastANI v.1.3 (55), while Roary v.3.13.0 was used for pangenome analysis (56). Secondary metabolite gene clusters, including bacteriocins and non-ribosomal peptides, were predicted using BAGEL4 (57) and antiSMASH v.7.0 (58).

### *In silico* analysis of pumicyclin A

The biosynthetic gene clusters of pumicyclin A were obtained from antiSMASH predictions and visualized using EasyFig with BLASTn alignment. Functional annotation of open reading frames was performed using BLAST (NCBI), and sequence identities were assessed using the SIAS server (Sequence Identity and Similarity) (59). Transmembrane domains were predicted with TMHMM v.2.0 (https://services.healthtech.dtu.dk/services/TMHMM-2.0/), while theoretical pI values were calculated using the ExPASy Compute pI/Mw tool (60).

The amino acid sequence of pumicyclin A was aligned with those of amylocyclicin and amylocyclicin CMW1 using MAFFT (https://www.ebi.ac.uk/jdispatcher/msa/mafft), and the alignments were visualized with Jalview v.10.0.5 (61). Secondary structure was predicted using PSIPRED v.4.0 (62), and a 3D model was generated using AlphaFold3 (63), then visualized in the Mol* Viewer (https://molstar.org/viewer/). Physicochemical properties, including molecular weight, net charge, pI, and hydrophobicity (GRAVY index), were calculated using ProtParam (64).

### Antibacterial activity assays

Antibacterial activity assays were performed using either bacteriocin-producing strains (overlay assays) or the isolated peptide (well diffusion assays). For activity testing with overlay assays, producer strains were cultured overnight in brain heart infusion (BHI) broth at 37°C with shaking at 160 rpm. A 2 μL aliquot of the culture was spotted onto BHI agar plates and air-dried under a biosafety cabinet. Plates were then incubated overnight at 37°C. The next day, the spotted plates were overlaid with soft agar (BHI/MRS, 0.75% agar) containing 0.3% of the indicator strain (Table S4). After 24 h of incubation under optimal conditions for the indicator, inhibition zones were measured using a digital caliper. Assays were conducted in triplicate.

For activity testing via well diffusion assays, pumicyclin A was isolated from its native producer, APC4183, using a modified freeze-thaw method (65). Briefly, overnight cultures of APC4183 were spread evenly onto MCM agar plates (10 g/L glucose, 2 g/L BHI, 1 g/L yeast extract, 2 g/L casein digest, 0.1 g/L magnesium sulfate) and incubated aerobically at 30°C for 24 h. The plates were then frozen at −80°C for 4 h, followed by thawing under sterile conditions. Agar from the plates was excised, and the eluate collected was passed through a 0.22 μm membrane filter to yield the FT-CFS. Antimicrobial activity of the FT-CFS was evaluated against indicator strains (Table S4) using the agar well diffusion assay described previously (66).

### Optimization of bacteriocin production

A one-factor-at-a-time approach (67) was used to identify the optimum carbon and nitrogen sources for bacteriocin production in APC4183. For carbon source optimization, LB agar (containing 10 g/L tryptone, 5 g/L yeast extract, 10 g/L NaCl, and 15 g/L agar) was supplemented with various carbon sources (30 g/L), including D-(−)-fructose, D-(+)-glucose, glycerol, lactose, D-(+)-mannose, D-(+)-galactose anhydrous, and sucrose. For nitrogen source optimization, tryptone and yeast extract were replaced with 15 g/L of alternatives such as beef extract, soy peptone, and BHI (porcine). Each formulation was tested by culturing APC4183 at 30°C for 24 h, after which the resulting FT-CFS was evaluated for antimicrobial activity against *L. innocua*. The most effective formulation was further supplemented with minerals and salts to enhance bacteriocin production (23). FT-CFS obtained from the optimized medium was tested against a broad panel of *Listeria* strains (Table 4) using well diffusion assays.

### Molecular cloning and mutagenesis

The gene cloning techniques were performed as previously described (68). Oligonucleotides used in this study were synthesized by Biolegio (Nijmegen, the Netherlands) (Table S5). DNA amplification, plasmid construction, and transformation were performed as described previously (69). *E. coli* DH5α was used as an intermediate host for constructs using vectors pMG36c, pDR111, and pTLR4 before transformation into their expression hosts, whereas pNZ8048c constructs were assembled directly in *L. lactis* NZ9000. All constructs were verified by sequencing (Macrogen Europe). Transformation of *B. subtilis* PG10, which harbors the competence genes (*comK*, *comS*) under the control of the mannitol-inducible promoter (P*_mtlA_*), was carried out following competence induction with 0.5% (wt/vol) mannitol (36). The successfully constructed strains were prepared as 20% glycerol (vol/vol) stocks and stored at −80°C for further experiments.

Mutagenesis was performed using the pMG36c-Pcn construct as a template, including both gene knockout of the bacteriocin biosynthetic gene cluster and site-directed mutagenesis of the precursor gene *pcnA*. Primers were designed based on the inverse PCR strategy to introduce the desired mutations. The procedures for DNA amplification, plasmid construction, and transformation followed the protocols described above. Successfully constructed *pcnA* mutants in *E. coli* were subsequently transformed into *B. subtilis* PG10, as previously outlined. All primers used in this study are listed in Table S5.

### Bacteriocin isolation and purification

Pumicyclin A was isolated from both native and recombinant producers. The native strain, APC4183, was subjected to the previously described freeze-thaw protocol. The obtained FT-CFS was fractionated by molecular weight using Amicon Ultra centrifugal filters (10 and 3 kDa cutoffs). Centrifugation was performed at 4,000 × *g* for 30 min at 4°C. In total, four size fractions were collected: >10 kDa, <10 kDa, 3–10 kDa, and <3 kDa. Solid-phase extraction was done with Strata C18-E columns (Phenomenex, UK). Specifically, the column was initially activated with HPLC-grade methanol, washed with HPLC-grade water, and then loaded with 6 mL of the FT-CFS sample. Unbound or weakly bound compounds were removed using a 30% ethanol-water wash. Stepwise elution was subsequently performed with 6 mL of isopropanol (10%–80%) containing 0.1% trifluoroacetic acid (TFA). All collected fractions were screened for antimicrobial activity against *L. innocua* using the well diffusion assay. Active fractions were subsequently concentrated via rotary evaporation and lyophilized for further analysis.

For heterologous expression in *B. subtilis* PG10, 5 mL of overnight culture was diluted into 100 mL of fresh LB broth and incubated at 37°C with aeration (200 rpm) for bacteriocin expression. After 4–6 h, cultures were centrifuged at 8,000 × *g* for 15 min, and the supernatant was collected for subsequent purification as previously reported (69). The mobile phase consisted of 0.2% TFA in water (solution A) and a 1:1 acetonitrile:isopropanol mixture (solution B). Fractions eluted with 20%, 50%, 80%, and 100% solution B were freeze-dried and reconstituted for further investigations.

### Bacteriocin identification and characterization

Purified fractions were analyzed using MALDI-TOF and tricine SDS-PAGE. For MALDI-TOF, 1 μL aliquots were analyzed as previously described (69). Where indicated, colony mass spectrometry was performed prior to purification following established protocols (21). For gel analysis, 20 μL peptide samples were mixed with 5 µL of loading buffer (containing 10% SDS, 0.5% Bromophenol blue, 50% glycerol, 250 mM Tris-HCl, pH 6.8) and incubated at 50°C for 30 min. Protein separation was carried out on a 16% separating gel alongside 5 µL of unstained low-range protein ladder (3.4–100 kDa, PageRuler, Thermo Fisher). Following separation, peptide bands were visualized using appropriate staining and destaining procedures as described previously (69).

Further SDS-PAGE analysis was conducted using 4–12% Bolt Tris-Gels (12-well). Briefly, 26 µL of active fractions (>10 kDa crude extract and IPA eluates), along with 5 µL of a prestained protein ladder (3–200 kDa, Invitrogen, Thermo Fisher Scientific), were run in duplicate on each half of the gel. Electrophoresis was carried out for 20 min using MES SDS running buffer (Invitrogen, Thermo Fisher Scientific) according to the manufacturer’s instructions. After electrophoresis, the gel was halved using a sterile scalpel: one half was used for anti-*Listeria* activity, and the other for peptide visualization. For activity testing, the gel was washed in 1% Triton X-100 for 1 h at room temperature with shaking, followed by three washes with sterile distilled water (20 min each). It was then placed in a sterile 92 × 16 mm petri dish and overlaid with 15 mL of 0.75% BHI sloppy agar inoculated with 0.5% *L. innocua* and incubated at 37°C overnight. The second half was rinsed three times with distilled water, stained with Imperial Protein Stain (Thermo Fisher Scientific) for 2 h, and destained overnight in water under gentle shaking.

### Aggregation assay

To test whether pumicyclin A aggregates due to hydrophobicity (causing retention above 10 kDa), a modified aggregation assay was used (70). FT-CFS was mixed with methanol at final concentrations of 0%, 25%, 50%, and 75% in a total volume of 5 mL. Each mixture was loaded into Amicon Ultra centrifugal filters (10 kDa MWCO, Millipore) and centrifuged at 5,000 rpm for 35 min. Both flow-through and retention fractions (<10 kDa and >10 kDa fractions) from each treatment were tested against *L. innocua* using the previously described well-diffusion assay. A 75% methanol control was included.

### Sequence homology search and analysis

Homologs of pumicyclin A were identified using BLASTP searches against the NCBI non-redundant protein database. The pumicyclin A core peptide sequence was used as the query, and hits were filtered using thresholds of ≥70% query coverage and ≥70% amino acid identity. Sequences annotated as circular bacteriocins within the uberolysin/carnocyclin family were compiled into Table S3.

## Data Availability

Sequenced data were deposited in GenBank under accession numbers QJIZ00000000.1 (MG52) and JBEPGF000000000JBEPGF000000000.1 (APC 4183).

## SUPPLEMENTAL MATERIAL

Supplemental material mbio.01188-26-s0001.docx Supplemental figures and tables.

ASM does not own the copyrights to Supplemental Material that may be linked to, or accessed through, an article. The authors have granted ASM a non-exclusive, world-wide license to publish the Supplemental Material files. Please contact the corresponding author directly for reuse.
