# Supplementary material for: Identification and characterization of pumicyclin A, a novel circular bacteriocin from Bacillus pumilus with a dispensable leader peptide
Source: mBio. 2026 Jun 24;17(8):e01188-26. doi: 10.1128/mbio.01188-26 (PMC13463998; doi:10.1128/mbio.01188-26)
Supplement: Supplementary file 1 [file mbio.01188-26-s0001.docx]

**Supplementary Materials**

**Identification and characterization of pumicyclin A, a novel circular bacteriocin from *Bacillus pumilus* with a dispensable leader peptide**

Fangfang Liu^1, 2^, Xiaohui Huang^1^, Paula M. O’Connor^3^, Felipe Miceli de Farias^1^, Colin Buttimer^1^, Colin Hill^1, 4^, Oscar P. Kuipers^2^, and R. Paul Ross^1, 3, 4*^

^1^APC Microbiome Ireland, University College Cork, Cork, T12 YT20 Cork, Ireland.

^2^Department of Molecular Genetics, University of Groningen, 9747 AG Groningen, the Netherlands

^3^Teagasc Food Research Centre, Moorepark, Fermoy, Co. Cork P61 C996 Co. Cork, Ireland.

^4^School of Microbiology, University College Cork, Cork, T12 Y337 Cork, Ireland.

^*^Address correspondence to R. Paul Ross, [p.ross@ucc.ie](mailto:p.ross@ucc.ie)

**Table S1**. Genome features of Bacillus pumilus APC4183 and Bacillus pumilus MG52.

| **Strain** | **Source** | **Total length (bp)** | **Contigs** | **GC content (%)** | **CDS** | **rRNA** | **tRNA** | **tmRNA** | **Accession N.** |
| --- | --- | --- | --- | --- | --- | --- | --- | --- | --- |
| *B. pumilus* APC4183 | soil | 3,676,682 | 35 | 41.6 | 3703 | 12 | 74 | 1 | JBEPGF000000000 |
| *B. pumilus* MG52 | soil | 3,803,730 | 47 | 41.6 | 3868 | 10 | 70 | 1 | QJIZ00000000.1 |

**Table S2.** (a) Anti-Listeria activity of size fractionated FT-CFS.

| **Size fraction** | |
| --- | --- |
| **Fraction** | **Activity** |
| Control (FT-CFS) | + |
| >10 | **+** |
| 3-10 | - |
| <10 | - |
| <3 | - |

“+” indicates active; “–” indicates inactive.

**(b)** Anti-Listeria activity of size fractionated FT-CFS-methanol mixture.

| **Methanol concentration** | **>10 kDa** | **<10 kDa** |
| --- | --- | --- |
| 0% | + | - |
| 25% | + | - |
| 50% | + | + |
| 75% | + | + |

**Control**: 75% methanol showed no anti-*Listeria* activity.

 “+” indicates active; “–” indicates inactive.

**Table S3.** Pumicyclin A variants across some other bacterial species (both Query Cover and Per. Ident above 70% from the NCBI database).

| Description | Taxon |  | Query Cover | E value | Per. ident | Accession |
| --- | --- | --- | --- | --- | --- | --- |
| MULTISPECIES: uberolysin/carnocyclin family circular bacteriocin [*Bacillus*] | *Bacillus* |  | 100% | 8.00E-32 | 100 | [WP_003214902.1](https://www.ncbi.nlm.nih.gov/protein/WP_003214902.1?report=genbank&log$=prottop&blast_rank=1&RID=ZN3ZTV1U013) |
| uberolysin/carnocyclin family circular bacteriocin [*Bacillus pumilus*] | *Bacillus pumilus* |  | 100% | 4.00E-31 | 98.44 | [WP_144485659.1](https://www.ncbi.nlm.nih.gov/protein/WP_144485659.1?report=genbank&log$=prottop&blast_rank=2&RID=ZN3ZTV1U013) |
| MULTISPECIES: uberolysin/carnocyclin family circular bacteriocin [*Bacillus*] | *Bacillus* |  | 100% | 1.00E-30 | 96.88 | [WP_025093875.1](https://www.ncbi.nlm.nih.gov/protein/WP_025093875.1?report=genbank&log$=prottop&blast_rank=3&RID=ZN3ZTV1U013) |
| MULTISPECIES: uberolysin/carnocyclin family circular bacteriocin [*Bacillus*] | *Bacillus* |  | 83% | 7.00E-21 | 94.34 | [WP_064498800.1](https://www.ncbi.nlm.nih.gov/protein/WP_064498800.1?report=genbank&log$=prottop&blast_rank=4&RID=ZN3ZTV1U013) |
| uberolysin/carnocyclin family circular bacteriocin [*Bacillus safensis*] | *Bacillus safensis* |  | 83% | 2.00E-20 | 94.34 | [WP_268437616.1](https://www.ncbi.nlm.nih.gov/protein/WP_268437616.1?report=genbank&log$=prottop&blast_rank=5&RID=ZN3ZTV1U013) |
| MULTISPECIES: uberolysin/carnocyclin family circular bacteriocin [*Bacillus*] | *Bacillus* |  | 75% | 6.00E-20 | 100 | [WP_226566725.1](https://www.ncbi.nlm.nih.gov/protein/WP_226566725.1?report=genbank&log$=prottop&blast_rank=6&RID=ZN3ZTV1U013) |
| uberolysin/carnocyclin family circular bacteriocin [*Exiguobacterium acetylicum*] | *Exiguobacterium acetylicum* |  | 83% | 3.00E-18 | 86.79 | [WP_308102445.1](https://www.ncbi.nlm.nih.gov/protein/WP_308102445.1?report=genbank&log$=prottop&blast_rank=7&RID=ZN3ZTV1U013) |
| uberolysin/carnocyclin family circular bacteriocin [*Bacillus xiamenensis*] | *Bacillus xiamenensis* |  | 78% | 4.00E-18 | 88 | [WP_008354999.1](https://www.ncbi.nlm.nih.gov/protein/WP_008354999.1?report=genbank&log$=prottop&blast_rank=8&RID=ZN3ZTV1U013) |
| MULTISPECIES: uberolysin/carnocyclin family circular bacteriocin [*Bacillus*] | *Bacillus* |  | 78% | 5.00E-18 | 88 | [WP_008343460.1](https://www.ncbi.nlm.nih.gov/protein/WP_008343460.1?report=genbank&log$=prottop&blast_rank=9&RID=ZN3ZTV1U013) |
| uberolysin/carnocyclin family circular bacteriocin [*Bacillus altitudinis*] | *Bacillus altitudinis* |  | 78% | 3.00E-17 | 86 | [WP_345911278.1](https://www.ncbi.nlm.nih.gov/protein/WP_345911278.1?report=genbank&log$=prottop&blast_rank=10&RID=ZN3ZTV1U013) |
| uberolysin/carnocyclin family circular bacteriocin [*Bacillus altitudinis*] | *Bacillus altitudinis* |  | 78% | 3.00E-17 | 86 | [WP_035702336.1](https://www.ncbi.nlm.nih.gov/protein/WP_035702336.1?report=genbank&log$=prottop&blast_rank=11&RID=ZN3ZTV1U013) |
| MULTISPECIES: uberolysin/carnocyclin family circular bacteriocin [*Bacillus*] | *Bacillus* |  | 78% | 5.00E-16 | 82 | [WP_256499084.1](https://www.ncbi.nlm.nih.gov/protein/WP_256499084.1?report=genbank&log$=prottop&blast_rank=12&RID=ZN3ZTV1U013) |
| MULTISPECIES: uberolysin/carnocyclin family circular bacteriocin [*Bacillus*] | *Bacillus* |  | 78% | 3.00E-15 | 80 | [WP_075612067.1](https://www.ncbi.nlm.nih.gov/protein/WP_075612067.1?report=genbank&log$=prottop&blast_rank=13&RID=ZN3ZTV1U013) |
| uberolysin/carnocyclin family circular bacteriocin [*Bacillus pumilus*] | *Bacillus pumilus* |  | 78% | 3.00E-15 | 80 | [WP_282067791.1](https://www.ncbi.nlm.nih.gov/protein/WP_282067791.1?report=genbank&log$=prottop&blast_rank=14&RID=ZN3ZTV1U013) |
| uberolysin/carnocyclin family circular bacteriocin [*Priestia flexa*] | *Priestia flexa* |  | 83% | 1.00E-13 | 77.36 | [WP_277717620.1](https://www.ncbi.nlm.nih.gov/protein/WP_277717620.1?report=genbank&log$=prottop&blast_rank=15&RID=ZN3ZTV1U013) |
| uberolysin/carnocyclin family circular bacteriocin [*Bacillus toyonensis*] | *Bacillus toyonensis* |  | 83% | 2.00E-13 | 73.58 | [WP_098227929.1](https://www.ncbi.nlm.nih.gov/protein/WP_098227929.1?report=genbank&log$=prottop&blast_rank=16&RID=ZN3ZTV1U013) |
| uberolysin/carnocyclin family circular bacteriocin [*Bacillus inaquosorum*] | *Bacillus inaquosorum* |  | 83% | 7.00E-13 | 71.7 | [WP_268290402.1](https://www.ncbi.nlm.nih.gov/protein/WP_268290402.1?report=genbank&log$=prottop&blast_rank=17&RID=ZN3ZTV1U013) |
| uberolysin/carnocyclin family circular bacteriocin [*Bacillus inaquosorum*] | *Bacillus inaquosorum* |  | 83% | 3.00E-12 | 71.7 | [WP_268290403.1](https://www.ncbi.nlm.nih.gov/protein/WP_268290403.1?report=genbank&log$=prottop&blast_rank=18&RID=ZN3ZTV1U013) |

**Table S4**. Growth conditions of bacterial indicators.

| **Indicator** | **Code** | **Growth media** | **Incubation temperature** |
| --- | --- | --- | --- |
| **Gram-positive strains** |  |  |  |
| *Lactobacillus delbrueckii* subsp. *bulgaricus* | LMG 6901 | MRS Difco | 37℃, anaerobic |
| *Leuconostoc mesenteroides* | KH-24 | MRS | 30℃ |
| *Listeria innocua* | UCC | BHI | 37℃ |
| *Listeria monocytogenes* | EDGe | BHI | 37℃ |
| *Clostridium perfringens* | EM 124 | RCM | 37℃, anaerobic |
| *Staphylococcus aureus* | DPC 5645 | TSB | 37℃ |
| *Staphylococcus epidermidis* | DSM 3095 | TSB | 37℃ |
| *Bacillus cereus* | DPC 6087 | TSB | 37℃ |
| **Gram-negative strains** |  |  |  |
| *Escherichia coli* | DPC 6054 | TSB | 37℃ |
| *Enterobacter cloaceae* | UCC | TSB | 37℃ |
| *Pseudomonas aeruginosa* | APC 2064 | TSB | 37℃ |

MRS: DeMan, Rogosa and Sharpe, BD Difco^TM^ Lactobacilli MRS; BHI: brain–heart infusion; RCM: reinforced clostridial media; TSB: tryptone soya broth. *Strains that were not specified with ‘anaerobic’ were incubated aerobically.

| **Table S5.** Oligonucleotides used in this study. | | |
| --- | --- | --- |
| **Variant** | **Primer** | **Sequence (5’→3’)** |
| **Construct** | | |
| pMG36c-Pcn | P01 | TTACAGTACAGCGACTATTTCTTTTCC |
|  | P02 | ATGACGAAAGCAACAGATTCTAAGT |
|  | P03 | ATCTGTTGCTTTCGTCATAATTAATCACCTCCTTTTGAAAAACTC |
|  | P04 | ATAGTCGCTGTACTGTAATTAAAGGCTCTAATAAGTTTGAGCAATAAC |
| pDR111-Pcn | P05 | CTTAGTCGACAGGAGATATACCATGACGAAAGCAACAGATTCTAAG |
|  | P06 | TTACAGTACAGCGACTATTTCTTTTCC |
|  | P07 | AGAAATAGTCGCTGTACTGTAAAGCTAGCCGCATGCAAG |
|  | P08 | GGTATATCTCCTGTCGACTAAGCTTAATTGTTATCCG |
| pNZ8048c-Pcn | P09 | ACTCACCAUGACGAAAGCAACAGATT |
|  | P10 | AGAAAGCTUACAGTACAGCGACTATTTC |
|  | P11 | AAGCTTTCUTTGAACCAAAATTAG |
|  | P12 | ATGGTGAGUGCCTCCTT |
| pTLR4-Pcn | P13 | AGGAGGCACUCAAAATGACGAAAGCAACAGATTCTA |
|  | P14 | ACCTCTGAAUTCCTTACAGTACAGCGACTATTTC |
|  | P15 | ATTCAGAGGUTTGATGACTTTG |
|  | P16 | AGTGCCTCCUTATAATTTATTTTG |
| **Knock-outs** | | |
| Δ *pcnA* | P17 | TCCTTCTTTATAAAACTTAGAATCTGTTGCTTTCGT |
|  | P18 | TTTTATAAAGAAGGAAAAGCAAAAGCTGC |
| Δ *pcnA’* | P19 | TCATTCCAAGGAAAAGCCAAGGCTG |
|  | P20 | TTTTCCTTGGAATGAGCCAAGATTTTTTGTTT |
| Δ *pcnB* | P21 | AAAATATGCATTACTATTTTCACGATTAC |
|  | P22 | AGTAATGCATATTTTAAATAGTTTATTTGTTTTATGCATAAG |
| Δ *pcnC* | P23 | TTTCTGATCATGATCAATAATAGATGGTTTTATTG |
|  | P24 | GATCATGATCAGAAAATACTTCAATCTTTTATGTATC |
| Δ *pcnD* | P25 | ACGAAGAGCATTATGAGTATAGGGGATGG |
|  | P26 | CATAATGCTCTTCGTACATTCTACAAATTCTAATC |
| Δ *pcnE* | P27 | TCATTGTTAGGAAAAGAAATAGTCGCTGTA |
|  | P28 | TTTTCCTAACAATGATGTTATTCTTGGACGTTGTTC |
| **Leader truncation** | | |
| Δ(-27-31) | P29 | CATAATTAATCACCTCCTTTTGAAAAACTC |
|  | P30 | TCTAAGTTTTATGCTTTATTATCGCTGTCAC |
| Δ(-21-31) | P29 | CATAATTAATCACCTCCTTTTGAAAAACTC |
|  | P31 | TTATCGCTGTCACTTCTTGCG |
| Δ(-11-31) | P29 | CATAATTAATCACCTCCTTTTGAAAAACTC |
|  | P32 | GTAGCTTTAGTGATTGGTAATGGTTC |
| Δ(-1-31) | P29 | CATAATTAATCACCTCCTTTTGAAAAACTC |
|  | P33 | ATTGCAGCAAATCTGGGTGTTAG |
| His-tag addition | P29 | CATAATTAATCACCTCCTTTTGAAAAACTC |
|  | P34 | CATCATCACCATCACCATACGAAAGCAACAGATTCTAAGTTTTATG |
| **Leader substitution** | | |
| L-1A | P35 | ATTGCAGCAAATCTGGGTGTTAG |
|  | P36 | CCAGATTTGCTGCAATCGCAGAACCATTACCAATC |
| L-1D | P35 | ATTGCAGCAAATCTGGGTGTTAG |
|  | P37 | CCAGATTTGCTGCAATATCAGAACCATTACCAATCAC |
| L-1K | P35 | ATTGCAGCAAATCTGGGTGTTAG |
|  | P38 | CCAGATTTGCTGCAATTTTAGAACCATTACCAATCAC |
| K-30A | P39 | CATAATTAATCACCTCCTTTTGAAAAACTC |
|  | P40 | ACGGCAGCAACAGATTCTAAGTTTTATGCT |
| D-27A | P39 | CATAATTAATCACCTCCTTTTGAAAAACTC |
|  | P41 | ACGAAAGCAACAGCTTCTAAGTTTTATGCTT |
| K-25A | P39 | CATAATTAATCACCTCCTTTTGAAAAACTC |
|  | P42 | ACGAAAGCAACAGATTCTGCGTTTTATGCTTTATTATCG |
| K-25A/F-24A/Y-23A | P39 | CATAATTAATCACCTCCTTTTGAAAAACTC |
|  | P43 | ACGAAAGCAACAGATTCTGCGGCTGCTGCTTTATTATCGCTGTCAC |
| **Bacteriocin derivative** | | |
| I1V | P44 | GTTGCAGCAAATCTGGGTGTTAG |
|  | P45 | CAAAGAACCATTACCAATCACTAAAGC |
| I1L | P46 | CTTGCAGCAAATCTGGGTGTTAG |
|  | P45 | CAAAGAACCATTACCAATCACTAAAGC |
| I1M | P47 | ATGGCAGCAAATCTGGGTGTTAG |
|  | P45 | CAAAGAACCATTACCAATCACTAAAGC |
| I1A | P48 | GCTGCAGCAAATCTGGGTGTTAG |
|  | P45 | CAAAGAACCATTACCAATCACTAAAGC |
| A2V | P49 | ATTGTTGCAAATCTGGGTGTTAGTACTGC |
|  | P45 | CAAAGAACCATTACCAATCACTAAAGC |
| A2I | P50 | ATTATTGCAAATCTGGGTGTTAGTACTGC |
|  | P45 | CAAAGAACCATTACCAATCACTAAAGC |
| A2L | P51 | ATTCTTGCAAATCTGGGTGTTAGTACTGC |
|  | P45 | CAAAGAACCATTACCAATCACTAAAGC |
| A2D | P52 | ATTGATGCAAATCTGGGTGTTAGTACTG |
|  | P45 | CAAAGAACCATTACCAATCACTAAAGC |
| A2K | P53 | ATTAAAGCAAATCTGGGTGTTAGTACTG |
|  | P45 | CAAAGAACCATTACCAATCACTAAAGC |
| A63V | P54 | GAAAAGCAAAAGCTGCTGTATTTTAATCTAAAAAAAAATCATC |
|  | P55 | AGCAGCTTTTGCTTTTCCTTC |
| A63I | P56 | GAAAAGCAAAAGCTGCTATTTTTTAATCTAAAAAAAAATCATCTC |
|  | P55 | AGCAGCTTTTGCTTTTCCTTC |
| A63L | P57 | GAAAAGCAAAAGCTGCTCTTTTTTAATCTAAAAAAAAATCATCTC |
|  | P55 | AGCAGCTTTTGCTTTTCCTTC |
| A63D | P58 | GAAAAGCAAAAGCTGCTGATTTTTAATCTAAAAAAAAATC |
|  | P55 | AGCAGCTTTTGCTTTTCCTTC |
| A63K | P59 | GAAAAGCAAAAGCTGCTAAATTTTAATCTAAAAAAAAATCATCTC |
|  | P55 | AGCAGCTTTTGCTTTTCCTTC |
| F64A | P60 | GAAAAGCAAAAGCTGCTGCTGCTTAATCTAAAAAAAAATCATC |
|  | P55 | AGCAGCTTTTGCTTTTCCTTC |
| F64W | P61 | GAAAAGCAAAAGCTGCTGCTTGGTAATCTAAAAAAAAATCATC |
|  | P55 | AGCAGCTTTTGCTTTTCCTTC |
| F64Y | P62 | GAAAAGCAAAAGCTGCTGCTTACTAATCTAAAAAAAAATCATC |
|  | P55 | AGCAGCTTTTGCTTTTCCTTC |

**Note:** primers P29, P39, P45 are 5′-phosphorylated.

**Figure S1.** Alignment of pumicyclin A and its smaller bacteriocin-like peptide encoded within its biosynthetic gene cluster.

**Figure S2.** Optimization for the pumicyclin A production

**Figure S3.** Bacteriocin production comparison between constructs with the addition of a 6xHis-tag and leader truncations

**Figure S4**. MALDI‑TOF MS analysis and antimicrobial activity of pumicyclin A produced with truncated leader peptides. MALDI‑TOF mass spectra of pumicyclin A from the wild‑type construct and variants containing progressively shortened leader peptides. Insets show corresponding antimicrobial activity assays. The detected masses were consistent with the expected mass of mature pumicyclin A across all constructs, with slight deviations. This indicates that an extended leader peptide is not required for bacteriocin maturation or activity. A complementary analysis providing clearer and more accurate mass spectra for the wild‑type construct and the 1‑amino‑acid leader variant is presented in **Figure S5**.

**Figure S5**. MALDI‑TOF MS analysis and antimicrobial activity of pumicyclin A produced by the wild-type construct and the 1-aa leader variant. The observed masses closely match the expected mass of mature pumicyclin A (~6218 Da). Insets display the corresponding antimicrobial activity assays. These results suggest proper maturation and activity in both constructs.

**Figure S6**. Leader peptide sequences of circular bacteriocins. At position −1, charged residues (highlighted in yellow) are frequently observed in group I circular bacteriocin leader sequences, whereas the N−1 residue is conserved in group II leader sequences. At position −2, long-leader circular bacteriocins (left) preferentially contain a small residue, while short-leader circular bacteriocins (right) tend to harbour a comparatively bulkier residue.

*Group I circular bacteriocins: enterocin AS-48 (AS-48), bacA (BacA), cerecyclin (CycA), pumilarin (PumA), amylocyclicin (AcnA), Amylocyclicin_CMW1 (AccA), enterocin NKR-5-3B (Ent53B), circularin A (CirA), uberolysin A (UblA), carnocyclin A (CclA), leucocyclicin Q (LcyQ), lactocyclicin Q (LycQ), garvicin ML (GarML), bacicyclicin XIN-1 (BccA), and aureocyclicin 4185 (AclA); Group II circular bacteriocins: acidocin B (AciB), gassericin A (GaaA), plantaricyclin A (PlcA), plantacyclin B21AG (PtcA), butyrivibriocin AR10 (BviA), and paracyclicin (PrcA).
